# MAML-residual transformer for few-shot prediction of targeted therapy response in NSCLC

**DOI:** 10.3389/fcell.2026.1866438

**Published:** 2026-07-30

**Authors:** Hua-Jun Lu, Guo-Chao Ren, Dao-Gui Chen, Guo-Xiao Lv, Ting Ying, Hui-Xin Qi, Jia-Dong Hua, Chao-Qun Wang

**Affiliations:** 1 Department of Oncological Radiotherapy, Affiliated Dongyang Hospital, Wenzhou Medical University, Dongyang, China; 2 School of Reliability and Systems Engineering, Beihang University, Beijing, China; 3 Department of Pathology, Affiliated Dongyang Hospital of Wenzhou Medical University, Dongyang, China

**Keywords:** few-shot learning, medical image analysis, model-agnostic meta-learning (MAML), non-small cell lung cancer (NSCLC), residual transformer

## Abstract

**Introduction:**

Non-small cell lung cancer (NSCLC) exhibits high heterogeneity, and the scarcity of annotated imaging data limits the robustness and generalization capability of conventional deep learning approaches, particularly in few-shot scenarios. Therefore, developing accurate and generalizable prediction models under limited sample conditions remains a critical challenge for precision oncology.

**Methods:**

In this study, we proposed a Model-Agnostic Meta-Learning_RT (MAML_RT) framework for predicting targeted therapy response in NSCLC. The framework integrates model-agnostic meta-learning with residual transformers. Multi-task meta-training was employed to learn highly generalizable parameter initializations, enabling rapid adaptation to new cohorts with limited labeled samples. Meanwhile, the multi-head self-attention mechanism of the residual transformer was utilized to model global correlations among radiomics features and capture long-range dependencies between diverse tumor phenotypic characteristics.

**Results:**

In a cohort of 300 patients, MAML_RT achieved an accuracy of 0.91 and an area under the receiver operating characteristic curve (AUC) of 0.93 for predicting targeted therapy efficacy. Under an extremely small-sample scenario (n = 50), the model maintained an AUC of 0.76, significantly outperforming the comparison models. Furthermore, on an independent external validation cohort, MAML_RT achieved an AUC of 0.85 and an accuracy of 0.88, demonstrating its cross-center generalization capability.

**Discussion:**

The proposed MAML_RT framework provides an effective solution for targeted therapy response prediction in NSCLC under limited labeled data conditions. By integrating meta-learning with residual transformer-based feature modeling, this approach improves model adaptability and generalization, offering potential support for clinical decision-making and individualized treatment stratification.

## 1 Introduction

NSCLC is the predominant histological subtype of lung cancer, accounting for approximately 85%–90% of all lung cancer cases. It is the leading cause of cancer-related deaths worldwide. According to 2022 global cancer statistics, NSCLC accounts for 2.48 million new cases and approximately 1.81 million deaths annually (Yuan et al., 2025). Biologically, NSCLC exhibits high molecular heterogeneity involving multiple driver gene mutations, including EGFR, KRAS, ALK, ROS1, and MET. These mutations serve as core drivers of tumorigenesis and progression while profoundly influencing individualized treatment responses and clinical outcomes (Wang et al., 2025). Epidemiological evidence further indicates that the risk of developing NSCLC and its mutation profiles exhibit significant racial and gender biases: Asian populations and non-smoking women show relatively higher prevalence rates, with EGFR mutations demonstrating a marked epidemiological trend among Asian patient cohorts (Huang et al., 2025). The aforementioned characteristics collectively reveal the high molecular and population-level heterogeneity of NSCLC, providing a crucial foundation for developing precision treatment strategies.

Based on the identified driver gene mutation profile, targeted therapy with tyrosine kinase inhibitors (TKIs) has become the standard treatment for specific subtypes of NSCLC. Multiple clinical studies have confirmed that drugs such as the EGFR inhibitor osimertinib, the ALK inhibitor alectinib, and the KRAS G12C inhibitor sotorasib can significantly prolong patients’ progression-free survival (PFS) and overall survival (OS) (Liu et al., 2025). For instance, osimertinib as first-line therapy achieves a median PFS of 18.9 months in EGFR-mutated NSCLC patients, while sotorasib demonstrates a 37.1% objective response rate (ORR) in KRAS G12C-mutated patients who have failed prior treatments (Yuan et al., 2025). However, prolonged treatment duration and the widespread emergence of resistance mechanisms significantly diminish the long-term benefits of targeted therapies. Primary resistance, such as that caused by KEAP1/STK11 co-mutations, and acquired resistance mediated by EGFR T790M mutations or MET amplification, can both lead to treatment failure and accelerated disease progression (Qian et al., 2025). To overcome the efficacy limitations of monotherapy, combination strategies integrating immune checkpoint inhibitors (ICIs) with TKIs have entered critical clinical exploration phases. While demonstrating potential synergistic antitumor effects in some patients, these approaches exhibit significant interindividual variability in clinical response and carry safety concerns such as cumulative toxicity risks (Zhang et al., 2024). This dual challenge of “uncertain efficacy” and “increased toxicity risk” makes the accurate identification of potential beneficiaries before or early in treatment a core issue requiring urgent resolution in precision medicine. Therefore, constructing predictive models with high reliability and strong generalization capabilities for achieving refined patient stratification and dynamic assessment of treatment response holds significant clinical value.

However, in practical modeling, constructing high-performance image prediction models remains constrained by both limited sample sizes and high annotation costs. This challenge is particularly pronounced in rare driver mutation subgroups, such as MET exon 14 skipping or NTRK fusions, where data scarcity is especially acute (Diebels and Van Schil, 2022). In recent years, artificial intelligence has been widely applied in industry (Liu et al., 2026; Yang et al., 2026; Pan et al., 2025; Ji et al., 2026; Li et al., 2026; Tong et al., 2026) and medicine due to its powerful capabilities in high-dimensional data representation and automatic feature extraction. However, in low-data-volume scenarios, the generalization performance of traditional deep learning models often deteriorates significantly (Bray et al., 2018). Although strategies like data augmentation and transfer learning partially alleviate the sample shortage issue, they remain significantly limited in feature robustness and cross-domain adaptability (Dharmawan et al., 2025). Meanwhile, traditional radiomics-based analysis methods, while offering interpretability advantages, exhibit insufficient stability when confronted with heterogeneous imaging data due to their heavy reliance on manual feature engineering and demand for large-scale annotated datasets (Helissey et al., 2022; Song et al., 2020; Dai et al., 2020). Existing research indicates that most deep learning models typically require over 500 cases to achieve clinically acceptable robustness levels. In cross-center validation, domain shifts caused by variations in scanning protocols and equipment often result in a 15%–30% decline in model performance (Wang et al., 2020; Attili et al., 2020). Therefore, against the backdrop of scarce samples, significant domain shifts, and cross-center applications becoming the norm, there is an urgent need to explore novel modeling paradigms that can overcome the traditional data dependency bottleneck of deep learning and maintain stable performance under few-shot conditions.

For the challenge of few-shot learning, meta-learning has emerged as a promising solution. The MMOSurv framework proposed by Wen and Li (2025) demonstrates that meta-learning can significantly improve the generalization capability of modeling high-dimensional biomedical data with few samples by sharing feature representations across tasks, while effectively mitigating the overfitting issues caused by sample scarcity. Meanwhile, the adversarial pre-training zero-shot meta-learning method developed by Wu and Bergman (2025) for table prediction tasks further demonstrates that combining pre-training mechanisms with meta-learning-based parameter initialization strategies can endow models with stronger cross-task and cross-domain transfer capabilities, providing a novel algorithmic optimization approach for small-sample medical modeling. Although the prototype network has been preliminarily validated for the EGFR mutation prediction task, the model’s performance is highly susceptible to fluctuations in imaging conditions (Wu et al., 2020). In contrast, Model-Agnostic Meta-Learning (MAML) offers distinct advantages in rapid task adaptation. However, when directly processing three-dimensional CT volume data, it often faces challenges of training instability and excessive computational overhead. To address this, Moghaddam et al. (2025) proposed a model-agnostic meta-learning framework that integrates multi-source data while optimizing feature selection and classifier training. Notably, when combined with a Transformer encoder, this framework achieved outstanding performance with a classification accuracy of 96.89%, outperforming traditional statistical methods and conventional deep learning models. Building upon this, Hu et al. (2023) proposed a MAML-based lung cancer diagnosis model (MLLCD) that fuses convolutional neural networks (CNNs) with Transformers to extract local and global features from pathological images. This model demonstrates rapid adaptation under few-shot conditions, achieving ROC values of 0.94 and 0.98 on the LSCC dataset under 5-shot and 20-shot configurations, respectively. Furthermore, Anandhi et al. (2024) proposed a diagnostic framework integrating image augmentation, segmentation, transfer learning, and few-shot classification to effectively mitigate data scarcity while enhancing diagnostic accuracy and efficiency.

Thanks to its powerful ability to model long-range dependencies and capture global feature interactions, the Transformer architecture has become the leading architecture for modeling high-dimensional data with few examples. In the field tabular and omics features, the TabPFN tabular foundation model proposed by Hollmann et al. (2025) has further refined the theoretical framework for few-shot learning in this architecture. Related studies have confirmed that the Transformer’s self-attention mechanism can adaptively uncover global correlations among multidimensional features, demonstrating excellent generalization performance in small-sample tabular data tasks. Despite these significant advantages, the Transformer architecture—with its vast number of parameters—is highly susceptible to the scarcity of medical samples and is prone to overfitting in small-sample scenarios, which severely limits its direct application in clinical modeling for lung cancer (Du et al., 2020; Zang et al., 2021). To overcome this bottleneck, numerous studies have explored modified Transformer models focused on lightweight and adapted designs. Kipkogei et al. (2021) constructed a clinical Transformer model that leverages attention mechanisms to analyze deep correlations between clinical and molecular features, while combining transfer learning to optimize modeling performance in small-sample settings. Ultimately, they achieved a C-index of 0.61, with the model results demonstrating good interpretability. Concurrently, Imran et al. (2024) designed a hybrid architecture combining CNNs and visual Transformers for the classification of lung pathology images. This approach accurately distinguished between normal lung tissue, lung adenocarcinoma, and lung squamous cell carcinoma, achieving a high classification accuracy of 0.988 on the LC25000 dataset. Building on this, Amidi et al. (2025) further integrated Momentum Contrast Learning (MoCo-V3) with the Transformer architecture to develop a novel deep learning framework for screening ALK/ROS1 fusion mutations in lung cancer. In a large clinical cohort, they achieved an AUC of 0.85 for ROS1 mutations and 0.84 for the AUC of the ALK mutation, fully validating the clinical value of the improved Transformer model in screening for targeted therapy targets in lung cancer.

Although meta-learning and Transformers each possess unique strengths, their synergistic potential in predicting targeted therapy efficacy for NSCLC remains understudied. Existing single-paradigm approaches struggle to simultaneously achieve cross-domain generalization capabilities and precisely characterize the microscopic phenotypes of the complex tumor microenvironment (TME). This limitation is further amplified in real-world clinical applications: high-performance models typically require 300–500 high-quality annotated samples to ensure training stability, while actual sample sizes for rare mutation subtypes often fall below 50 cases (Yoshioka et al., 2019). Furthermore, during multi-center deployment, domain shift frequently causes a 15%–30% decline in model performance (Yu et al., 2013). Consequently, this structural contradiction between “high-performance requirements” and “scarce annotated samples” urgently demands new methodological breakthroughs.

Based on this, the primary objective of this study is to identify imaging phenotypic information associated with EGFR-TKI efficacy through medical image mining, thereby providing a new non-invasive assessment method for predicting treatment response. Although the current study has not yet directly integrated pathological, genomic, and molecular resistance data, previous research has shown that the tumor heterogeneity, textural complexity, and spatial distribution characteristics reflected by radiomic features may be closely related to biological processes such as changes in the tumor microenvironment (Guo et al., 2025), hypoxia, the formation of necrotic areas (Cook et al., 2013), and clonal evolution (Wang et al., 2023)—processes that are considered key mechanisms underlying acquired resistance to EGFR-TKIs (Qie et al., 2026). Therefore, imaging-based treatment response prediction models not only offer the advantages of being non-invasive and reproducible but also have the potential to identify potential imaging biomarkers associated with resistance mechanisms at the molecular level.

To address these challenges and achieve the aforementioned scientific objectives, this study proposes a novel computational medicine integrated prediction framework named MAML_RT, designed to simultaneously overcome the issues of limited imaging samples and restricted cross-domain generalization in non-small cell lung cancer prediction. By synergistically integrating MAML with Residual Transformers, this framework achieves robust performance under data-scarce conditions. The core innovations and biological mapping of MAML_RT are manifested at the following two complementary levels:

1. At the learning strategy level: MAML is employed to learn parameter initializations with strong generalization potential. This design enables the model to rapidly adapt to new NSCLC patient cohorts using only a small number of labeled samples and a few gradient updates, thereby substantially reducing reliance on large-scale training data.
2. At the feature modeling level: The RT takes CT-based quantitative tumor features extracted via pyradiomics as input and models global correlations among high-dimensional features through a multi-head self-attention mechanism. This approach effectively captures long-range dependencies between diverse tumor phenotypic features and addresses the limitations of conventional convolutional or fully connected architectures in representing complex feature interactions.

Overall, the proposed framework provides a clinically deployable and sample-efficient solution for predicting targeted therapy efficacy in NSCLC, while maintaining a balance between predictive accuracy, generalization capability, and practical utility.

## 2 Materials and methods

**FIGURE 1:**
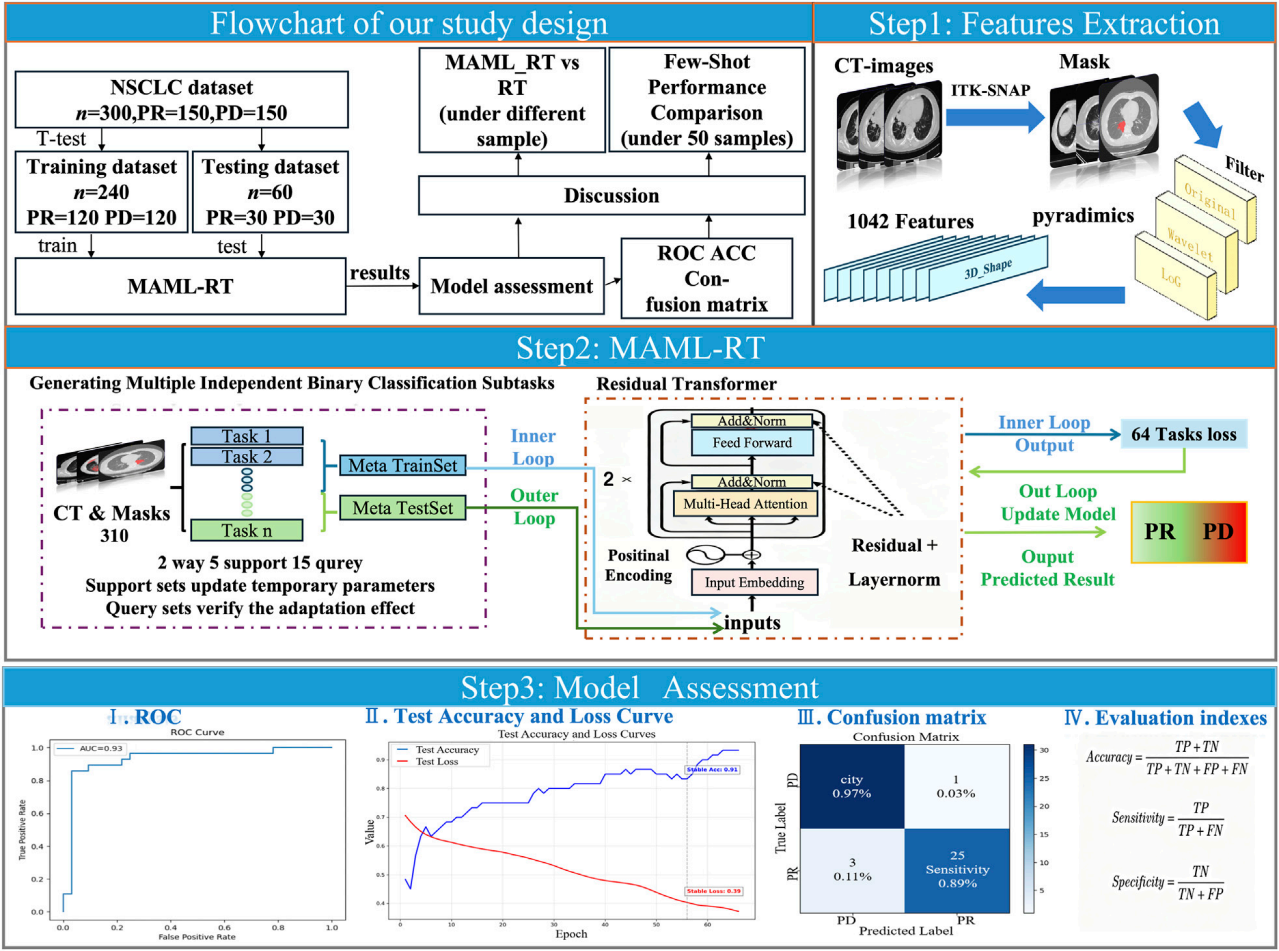
Overall flowchart of this study. The research process primarily consists of three stages: (1) Feature Extraction: Tumor segmentation of CT images using ITK-SNAP, with 1042 radiomics features extracted via pyradiomics; (2) MAML-RT: Generating multiple independent binary classification few-shot tasks within the MAML framework, incorporating a residual Transformer architecture, and jointly optimizing model parameters through inner and outer loops; (3) Model Evaluation: Comprehensive assessment of model performance using ROC curves, accuracy versus loss curves, confusion matrices, and multiple evaluation metrics.

Figure 1 shows the flowchart of proposed MAML_RT model for prognosis prediction and the detailed implementation steps including tumor segmentation, feature extraction, model construction and model evaluation.

### 2.1 Data collection

This retrospective study collected chest CT imaging data from 300 patients with EGFR-mutated NSCLC at Dongyang People’s Hospital (Approval No. is Dongrenyi 2024-YX-245), encompassing both non-contrast and contrast-enhanced CT examinations. The age distribution in this study cohort spanned a wide range: 7% were under 50 years old, 22% were 50–60 years old, 38% were 60–70 years old, 18% were 70–80 years old, and 15% were 80 years old or older. The age of onset in this cohort was primarily concentrated in the 60–70 age range (mode: 38%). This distribution closely aligns with the median age of onset (approximately 65–70 years) reported in large-scale real-world non-small cell lung cancer (NSCLC) registry studies both domestically and internationally, effectively covering the primary high-incidence age groups for NSCLC and thus demonstrating good representativeness across age groups (Sekine et al., 2020; Jeon et al., 2022; Debieuvre et al., 2013). Regarding gender distribution, male patients accounted for 27% of the study cohort, while female patients accounted for 73%. Although the proportion of female patients is significantly higher than that in traditional population-based lung cancer registries in Europe, the United States, and East Asia (Sekine et al., 2020; Jeon et al., 2022; Debieuvre et al., 2013), this characteristic closely aligns with the latest epidemiological trends in non-small cell lung cancer in China in recent years—namely, the continuous rise in lung cancer incidence among Chinese women (especially non-smoking women) and a trend toward younger onset. At the same time, given that this study focuses on predicting the efficacy of targeted therapies such as EGFR-TKIs, and considering that driver gene mutations (such as EGFR) are naturally highly enriched in female NSCLC patients in China, this has led to a clinically reasonable, structural increase in the proportion of female patients enrolled to receive targeted therapy (Zhang et al., 2018). Consequently, the demographic structure of this cohort accurately reflects the real-world characteristics of a specific subgroup of NSCLC patients receiving targeted therapy in contemporary China.

All enrolled patients received standardized EGFR-TKI targeted therapy, including first-generation (Gefitinib, Icotinib) or third-generation (Osimertinib) agents. To focus on constructing a universal predictive model for treatment response, this study treated TKIs of different generations as a unified targeted intervention variable. Treatment response was assessed based on imaging follow-up results and strictly adhered to the Response Evaluation Criteria in Solid Tumors (RECIST). Specifically, cases demonstrating a significant reduction in primary lesions, nodules, or enlarged lymph nodes were defined as partial response (PR); cases exhibiting lesion enlargement or new lesions were defined as progressive disease (PD).

The meta-training in this study was conducted within a single-center cohort (Dongyang People’s Hospital, 300 cases). Each meta-task was constructed by randomly sampling support sets and query sets from the training set via episodic resampling. Subsequently, we introduced an independent external validation set (from Cancer Prevention and Treatment Institute of Chengdu, Department of Oncology, Chengdu Fifth People’s Hospital, 17 cases) for zero-shot evaluation.

### 2.2 Tumor segmentation

**FIGURE 2:**
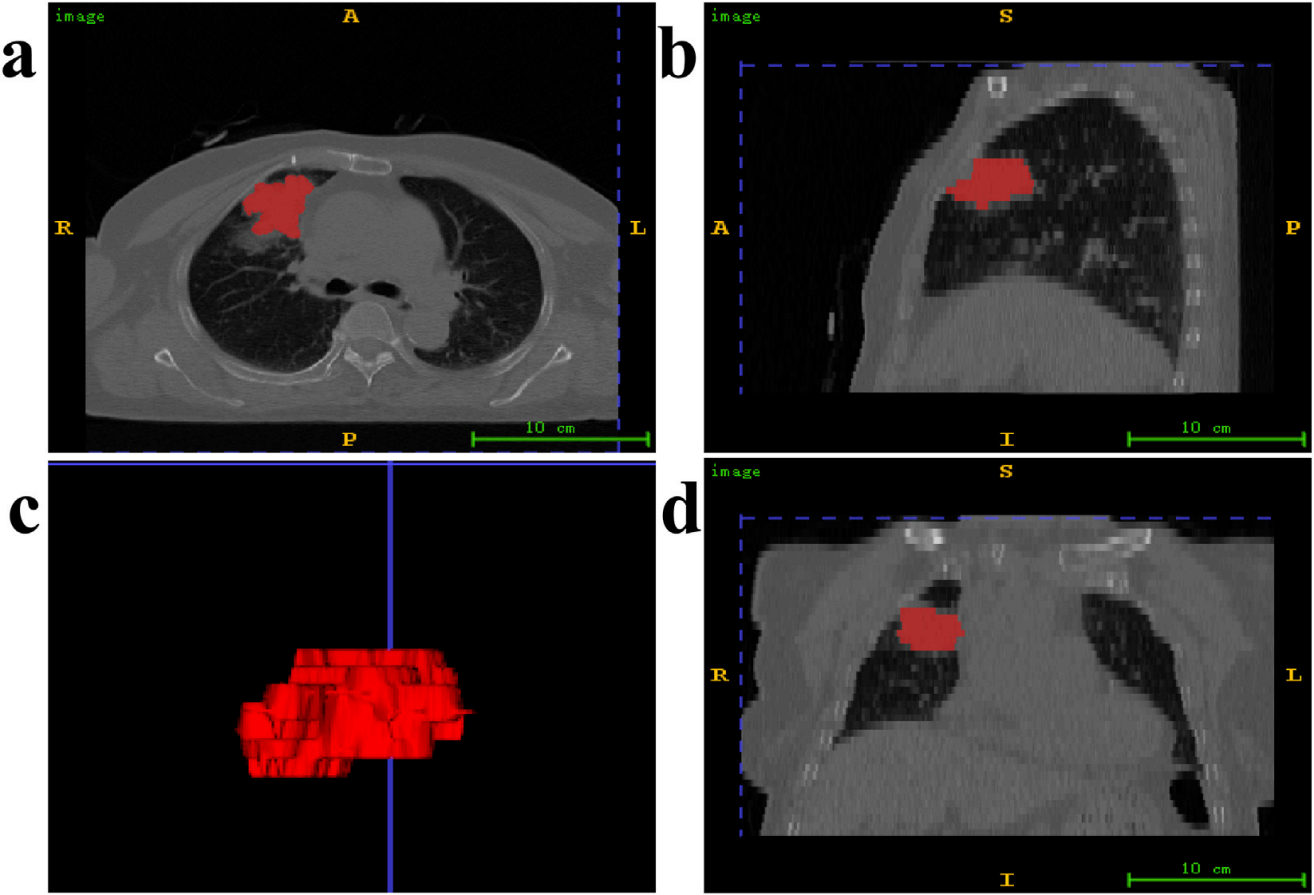
A CT image with the mask overlay for tumor segmentation: **(a)** axial view, **(b)** sagittal view, **(c)** 3D view, **(d)** coronal view.

All tumor regions on CT scan images are manually segmented by experienced radiologists. During segmentation, the overall lung fields were first reviewed to identify suspicious abnormalities. Radiology reports and clinical notes were referenced to refine lesion localization. Morphological and radiologic characteristics, such as maximum tumor diameter, lobulation, pleural indentation, spiculation, burr-like protrusions, bronchial air sign, cavitation, vacuole sign, calcification, and presence of hilar/mediastinal lymphadenopathy, were considered. The segmentation process is shown in Figure 2.

Each segmentation mask was exported and stored alongside the corresponding CT volume to facilitate subsequent review and reproducibility. All segmentations underwent secondary verification by senior thoracic radiologists. To reduce scanner- and patient-related heterogeneity, all CT volumes were resampled to a uniform spatial resolution prior to analysis, ensuring consistent voxel dimensions for downstream feature extraction and model training.

### 2.3 Feature extraction

**FIGURE 3:**
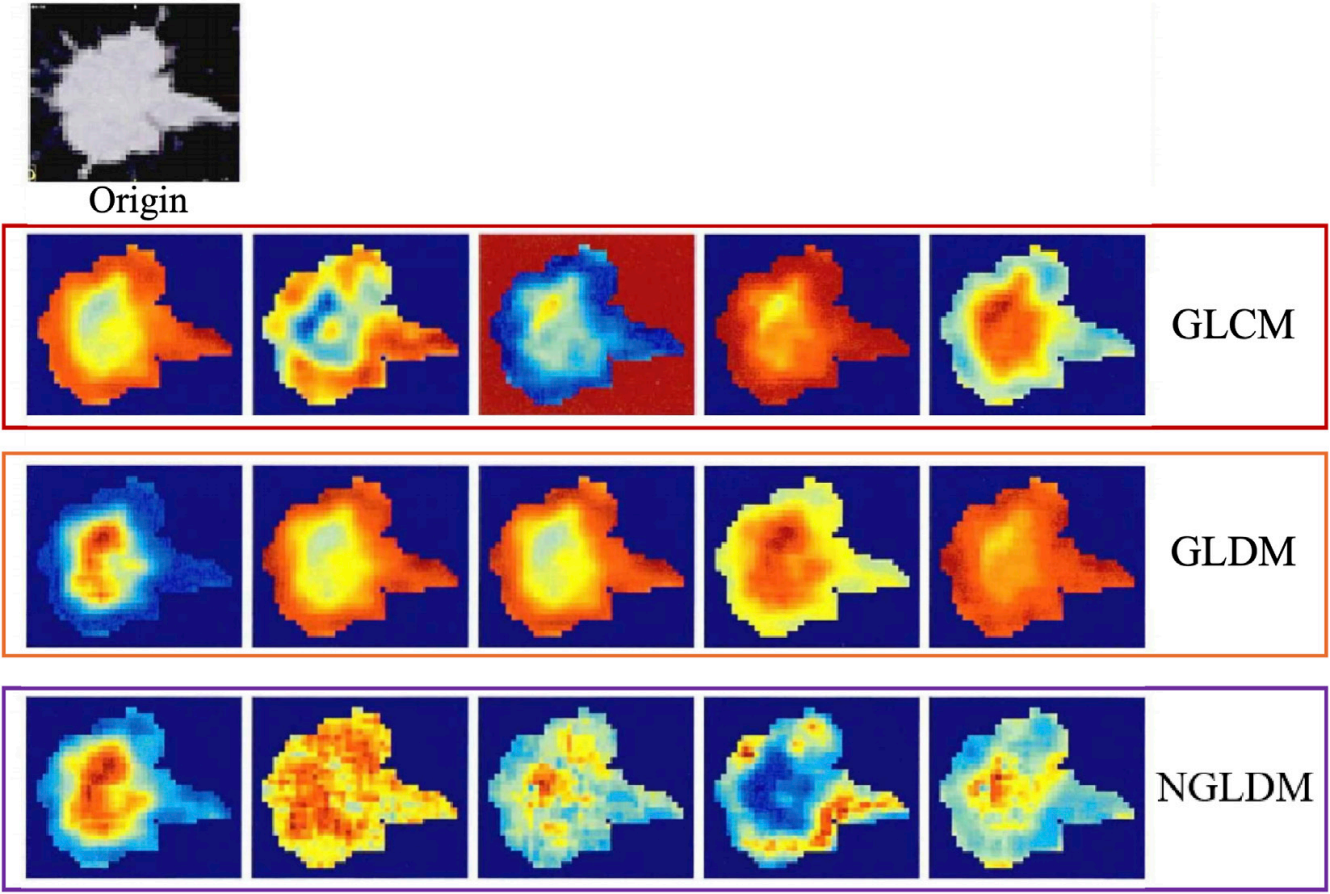
Original image and extracted CT grayscale features.

In evaluating the efficacy of targeted therapies for non-small cell lung cancer, traditional deep learning models and classical statistical methods often face challenges such as underutilization of high-dimensional features, insufficient exploration of complex feature interactions, and limited model stability, thereby restricting their clinical utility (Qi et al., 2026). To overcome these limitations and further explore the potential biological information embedded in non-invasive medical images, this study employs the open-source quantitative computing platform PyRadiomics to extract radiomic features from chest CT images, aiming to comprehensively characterize the heterogeneous features within tumors. To achieve a multi-scale, multi-dimensional characterization of lesion phenotypes, the feature extraction process not only relied on the original CT sequences but also encompassed edge enhancement using Gaussian Laplacian filters (LoG) at different σ levels and eight frequency subband images generated through three-dimensional discrete Wavelet Transform decomposition. Through these image transformations, the study extracted a total of 1,042 quantitative radiomics features. These encompass morphological features quantifying lesion geometry, first-order statistical features describing voxel gray-level intensity distributions, and texture features for deep characterization of spatial organization. Specifically involved are Gray-Level Co-occurrence Matrix (GLCM, seeing Figure 3), Gray-Level Long-Range Matrix (GLRLM), Gray-Level Size-Dependent Matrix (GLSZM), Neighborhood Gray-Level Difference Matrix (NGTDM), and Gray-Level Dependent Matrix (GLDM).

### 2.4 MAML_Residual transformer framework

Predicting treatment response in EGFR-mutated NSCLC is inherently a few-shot learning problem, given the biological heterogeneity across patients and the limited availability of labeled treatment-specific imaging data. To address this, we propose a predictive framework that integrates MAML with a Residual Transformer architecture.

#### 2.4.1 Model-agnostic meta-learning (MAML)

**FIGURE 4:**
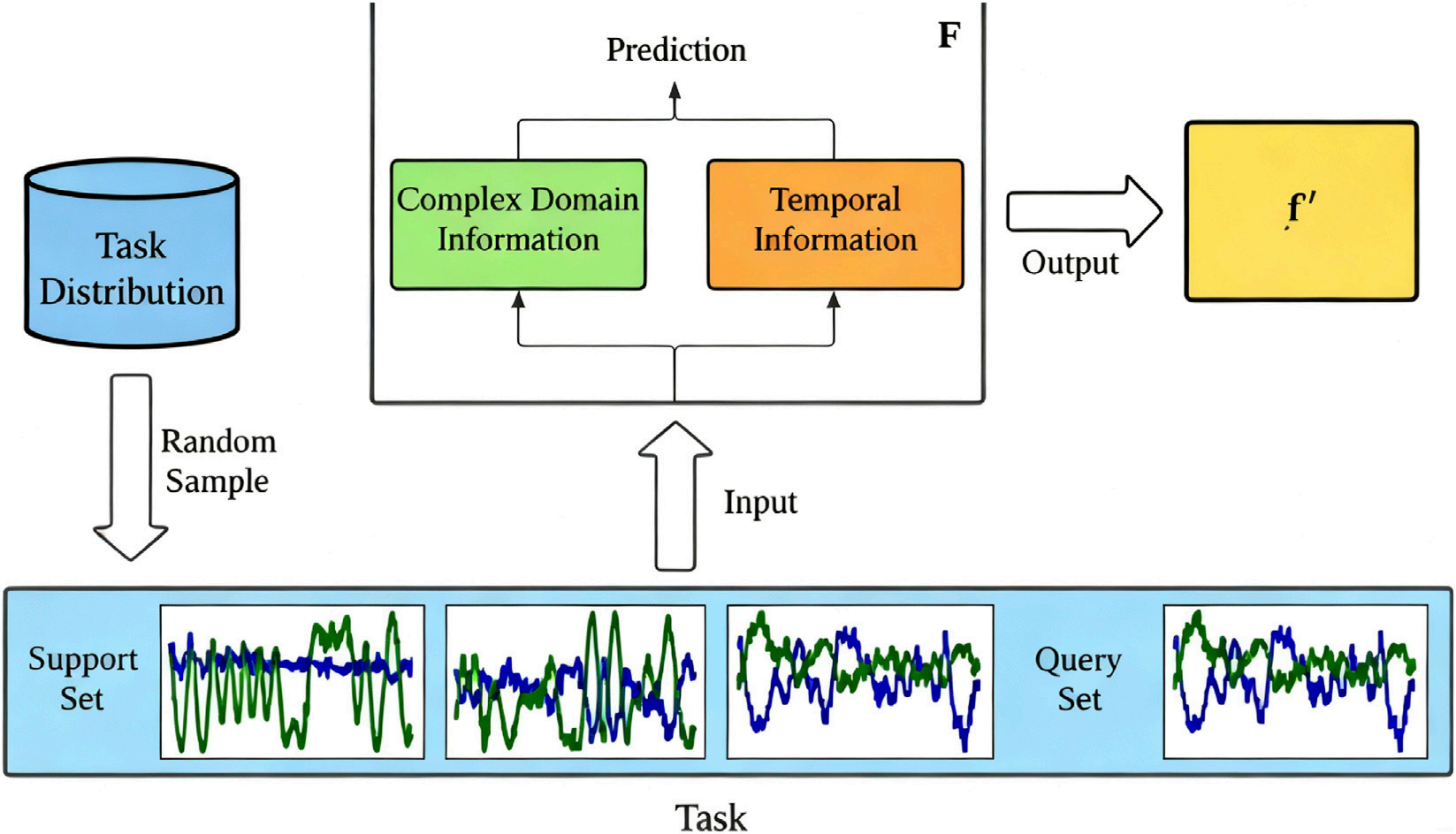
Schematic illustration of task construction and inference pipeline in meta-learning.

Meta-learning aims to endow models with the capabilities of cross-task transfer and rapid adaptation, and its core idea is “learning to learn”. As illustrated in Figure 4, traditional deep learning typically relies on a large amount of data with the same distribution to train fixed parameters. In contrast, meta-learning constructs a multi-task training framework, enabling models to extract learning strategies applicable to new tasks from a series of related tasks. Thus, the model can still quickly optimize its performance in scenarios with few-shot data or changes in task distribution.

The core idea of MAML lies in learning a model parameter initialization with strong transferability, enabling the model to rapidly adapt to new patient subgroups or prediction tasks with minimal gradient updates. During training, MAML employs a dual-loop optimization mechanism: in the inner loop, the model performs several gradient updates based on a small support set for a specific task, simulating rapid adaptation to new tasks; Subsequently, in the outer loop phase, the model’s performance is evaluated using the corresponding query set (Peng et al., 2024; Liu et al., 2020; Paeedeh et al., 2024) after the inner loop updates. Through backpropagation, the initial parameters are updated, thereby continuously optimizing the model’s generalization capability across tasks:

1. Inner-loop (Task-Specific Rapid Adaptation):

During meta-training, for each sampled task $\tau_{i}$ , the model parameters θ are temporarily adapted using one or several gradient descent steps on the task-specific support set $S_{i}$ , resulting in the task-adapted parameter set $\theta_{i}^{\prime }$ . See Equation 1.

$$\theta_{i}^{\prime }=\theta -\alpha \nabla_{\theta }{ϒ}_{S_{i}}\left(f_{\theta }\right)$$ (1)

Here, α denotes the inner-loop learning rate, and ${ϒ}_{S_{i}}\left(f_{\theta }\right)$ represents the loss computed on the support set. This step emulates the rapid adaptation of the model to a new NSCLC patient subgroup.

2. Outer-loop (Meta-Optimization):

As shown in Equation 2, the meta-learner then evaluates the performance of the adapted parameters $\theta_{i}^{\prime }$ on the query set $Q_{i}$ of the same task. The objective is to optimize the initial parameters θ such that the expected generalization error after adaptation across all tasks is minimized.

$$\min_{\theta }{Ε}_{\tau_{i}∼p\left(\tau \right)}\left[{ϒ}_{Q_{i}}\left(f_{\theta_{i}^{\prime }}\right)\right]\approx \min_{\theta }\frac{1}{B}\sum_{i=1}^{B}{ϒ}_{Q_{i}}\left(f_{\theta_{i}^{\prime }}\right)$$ (2)

Here, B denotes the number of tasks within a meta-batch. The meta-parameters θ are then updated by backpropagating through the inner-loop adaptation process (which typically involves second-order derivatives), thereby effectively learning an initialization that is highly amenable to rapid fine-tuning, see Equation 3:

$$\theta \leftarrow \theta -\beta \nabla_{\theta }\sum_{i=1}^{B}{ϒ}_{Q_{i}}\left(f_{\theta_{i}^{\prime }}\right)$$ (3)

Here, β denotes the meta-learning rate.

In our context, a task is defined as a predictive problem corresponding to a specific patient subgroup or a distinct molecular biomarker profile. The support set $S_{i}$ consists of a small number of annotated CT studies from patients within that subgroup, whereas the query set $Q_{i}$ contains additional studies from the same subgroup that are used to evaluate performance during meta-training.

#### 2.4.2 Backbone architecture: residual transformer

Although MAML provides a powerful learning algorithm, the selection $f\left(\theta \right)$ of the base model is still crucial. We adopt the Residual Transformer as the backbone network, as it has proven to be efficient in capturing multi-scale spatial dependencies in images—a feature that plays a key role in understanding the complex tumor morphology in CT volumes and the interaction between tumors and the surrounding lung parenchyma.

The input CT volume is first processed by a patch embedding layer: $\left\{X_{p}|p=1,...,N\right\}$ . This layer divides the image into a sequence of non-overlapping image patches, and each patch is then projected into a D-dimensional embedding space. Additionally, positional encoding is added to preserve the spatial information of the image.

The encoder consists of L hierarchical stages. Each stage starts with a Multi-Head Self-Attention (MHSA) mechanism, which allows each image patch to attend globally to all other patches, thereby dynamically aggregating the most relevant contextual information at each position, refer to Equation 4.

$$Attention\left(Q,K,V\right)=soft\max\left(\frac{QK^{T}}{\sqrt{d_{k}}}\right)V$$ (4)

The query (Q), key (K), and value (V) matrices are all obtained by projecting the input feature sequence through linear mappings. The self-attention mechanism uses the similarity calculation between Q and K to perform a weighted sum over V. This process models global dependencies between different positions in the sequence. Multi-head self-attention performs the above operations in parallel across h attention heads, enabling the model to simultaneously capture multi-scale and multi-semantic feature information across different representational subspaces. Subsequently, the attention outputs undergo a nonlinear feature transformation through a Feed-Forward Network (FFN). Residual connections are introduced both within the self-attention layer and outside the FFN sublayer to enhance gradient propagation stability and preserve the original feature representations.

#### 2.4.3 Collaborative integration: MAML and residual transformer

The high heterogeneity of NSCLC often renders models trained solely on general population distributions ineffective for new patients with rare molecular backgrounds or unique tumor phenotypes. This study utilizes MAML not only as a fine-tuning strategy but also as a parameter space structure for the meta-trained residual transformer. Here, model initialization is designed to adapt to variations in inter-patient distributions. The outer-loop optimization is not only about finding one optimal starting point. It aims to guide parameters to converge within a region of strong transferability. In this region, patient-specific models can achieve high performance with minimal gradient updates.

This meta-learning process requires more from the parameter space of the base model. The residual transformer, with its self-attention and hierarchical design, learns spatially aware, semantically rich, and continuous features from 3D CT data. This creates a smooth, well-structured parameter space. With this foundation, MAML learns to adapt these image representations to treatment response prediction. As a result, the model is robust to small-sample distribution shifts in new patients.

The complete end-to-end workflow of our proposed framework is as follows:

1. Meta-Training Phase:

A task distribution T is constructed from the base dataset of NSCLC patients with known treatment outcomes. Each task $T_{i}$ corresponds to a specific patient subgroup and is formed by randomly sampling a small support set $S_{i}$ and a query set $Q_{i}$ . Given the current meta-parameters θ , an inner-loop adaptation is performed for each task by applying gradient descent on the support set, yielding task-specific adapted parameters $\theta_{i}^{\prime }$ . Subsequently, in the outer-loop optimization, the meta-parameters θ are updated by minimizing the aggregated loss of all adapted models $\theta_{i}^{\prime }$ evaluated on their corresponding query sets $Q_{i}$ , thereby promoting generalization across tasks.

2. Meta-Testing Phase:

During the meta-testing phase, the framework is applied to a previously unseen patient, which is treated as a new task $T_{new}$ . A small support set $S_{new}$ , consisting of pre-treatment CT slices from this patient, is provided to perform inner-loop adaptation using the meta-learned initialization θ . This adaptation produces patient-specific parameters $\theta_{\text{new}}^{\prime }$ , which are then used to predict treatment outcomes for other data samples or future scans of the same patient.

The overall objective function during meta-training can be concisely formulated as shown in Equation 5:

$$\begin{array}{l} \theta^{*}=\arg\min_{\theta }{Ε}_{\tau_{i}∼p\left(\tau \right)}\left[{ϒ}_{Q_{i}}\left(f_{\theta_{i}^{\prime }}\right)\right]\\ =\text{rg}\min_{\theta }{Ε}_{\tau_{i}∼p\left(\tau \right)}\left[{ϒ}_{Q_{i}}\left(f_{\theta -\alpha \nabla_{\theta }{ϒ}_{S_{i}}\left(f_{\theta }\right)}\right)\right] \end{array}$$ (5)

This formula highlights the bilevel optimization nature of MAML, where the loss on the query set is a function of the parameters that have been optimized on the support set.

## 3 Results

**FIGURE 5:**
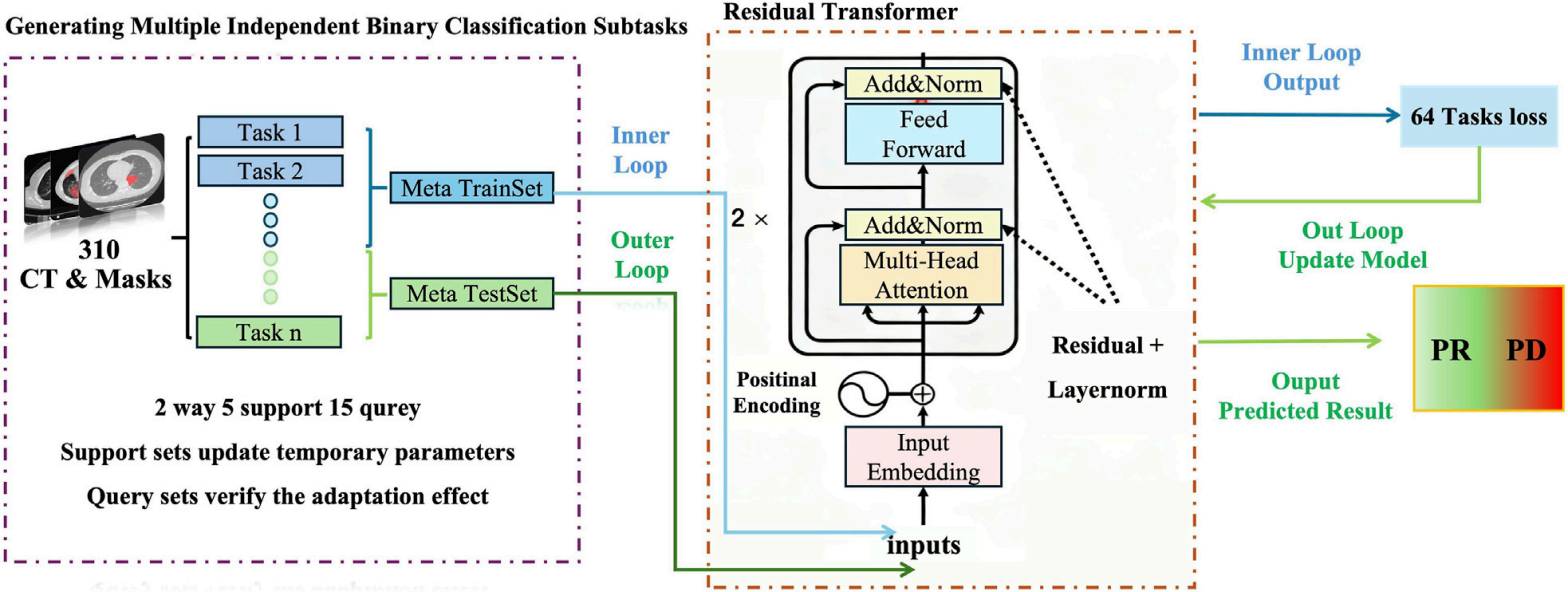
Framework Diagram of the MAML_RT Model. This framework achieves meta-learning training by constructing multiple independent binary classification few-shot tasks from CT images and their segmentation masks. The backbone network adopts a residual Transformer architecture comprising multi-head attention modules, feedforward networks, and residual connections with layer normalization to enhance feature modeling capability and stability.

Figure 5 shows the complete model framework of the MAML_RT model. Based on MAML’s data layer construction approach, we divided 300 NSCLC samples into an original training set and a meta-test set, which are used for model training and performance verification respectively. Meanwhile, the samples were divided into multiple independent binary classification subtasks, and each subtask consists of 2*(5 + 15) = 40 samples. Here, “2” represents the two categories of NSCLC samples: those with effective treatment (Partial Response, PR) and those with ineffective treatment (Progressive Disease, PD); “5” denotes the support set, which is used for model training and updating temporary parameters in the inner loop independent of meta-learning; “15” stands for the query set, which is used to verify the aforementioned training performance and output subtask loss for subsequent model parameter updates. Each inner-loop training involves 64 subtasks; the number of inner-loop steps is 1, referring to the number of steps for updating temporary parameters using the support set within each few-shot task; the learning rate of the inner-loop SGD optimizer is 0.01. The outer loop updates the global “meta-parameters” based on the query set loss of multiple tasks and verifies the model’s predictive ability for the targeted therapy effect of NSCLC on the test set. The number of meta-training epochs is 400; the number of first-order approximation epochs is 100, meaning that first-order approximation is used in the first 100 epochs to accelerate the gradient calculation of MAML; the learning rate of the meta-learning outer-loop Adam optimizer is 1e-4; gradient clipping is set to 1.0 to limit the gradient norm and prevent gradient explosion; the CosineAnnealingLR scheduling is configured with T_max = 400 and eta_min = 1e-6, where the learning rate decays periodically with epochs to improve training stability; the early stopping patience is 50, i.e., if the validation set AUC does not improve for 50 consecutive epochs, training is stopped to avoid overfitting.

As shown in the Residual Transformer module, this module consists of two Transformer encoder layers. Each layer is equipped with six multi-head attention heads (to capture global dependencies among features) and a 192-dimensional feedforward network (to enhance nonlinear representations). The input NSCLC tumor features are mapped to an embedding space with embed_dim = 96, providing the Transformer with a foundational representation. Concurrently, the Transformer’s output is summed with the embedding layer’s output via a residual connection, followed by layer normalization to mitigate vanishing gradients and improve the training stability of the deep network. Finally, the module consists of fully connected layers with a dimensionality reduction of “96→48→2,” a ReLU activation function, and Dropout, ultimately outputting the probability of binary classification (PR/PD).

It should be noted that the 1,042 radiomic features extracted in this study exceed the sample size (300 cases). This high-dimensional, small-sample configuration carries potential risks of overfitting, feature redundancy, and model instability. To address this issue, the original model design incorporated multiple explicit and implicit regularization strategies. Regarding explicit regularization, a dropout rate of 0.3 was applied after the linear layers of the Residual Transformer, and gradient clipping (capped at 1.0) was used in the outer meta-optimization layer to prevent gradient explosion. During training, an early stopping mechanism was employed (training stops if the validation set AUC does not improve for 50 consecutive epochs), and cosine-cooled learning rate scheduling was adopted to ensure stable convergence. Regarding implicit regularization, the meta-learning framework employs multi-task dual-loop optimization to force the model to learn transferable common patterns across tasks, thereby avoiding getting trapped in high-dimensional noise specific to a single task. Additionally, the multi-head self-attention mechanism in the Residual Transformer, together with layer normalization and residual connections, inherently suppresses overfitting.

**FIGURE 6:**
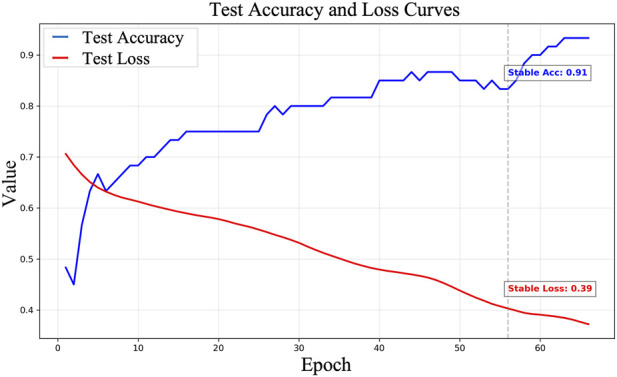
Curves of accuracy and loss for NSCLC targeted therapy efficacy prediction.

Figure 6 shows the curves of Test Accuracy and Test Loss of the NSCLC targeted therapy efficacy prediction model based on MAML_RT changing with training epochs. The blue curve represents Test Accuracy, which fluctuates slightly in the early stage of training but shows a continuous upward trend overall, eventually stabilizing at approximately 0.91 around the 60th Epoch. This result indicates that even in the few-shot scenario, the model can still achieve highly accurate prediction of NSCLC targeted therapy efficacy. The red curve represents Test Loss, which decreases continuously as the number of training epochs increases and finally converges to 0.39. This demonstrates that the model’s prediction error is constantly reducing, with excellent convergence, and it possesses strong fitting and generalization capabilities in few-shot learning tasks.

**FIGURE 7:**
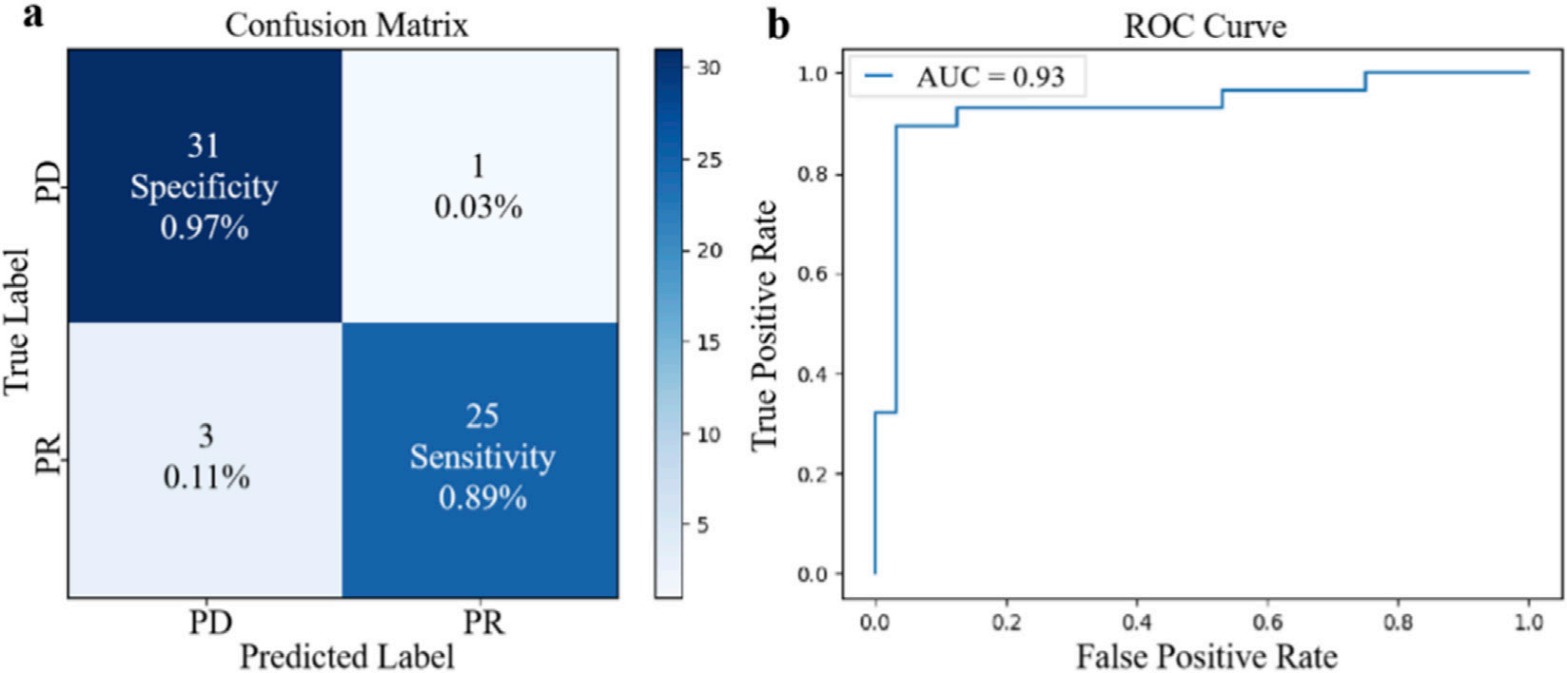
Prediction Results of NSCLC Targeted Therapy Efficacy: **(a)** Confusion Matrix, **(b)** ROC Curve.

Figure 7 presents the classification performance evaluation results of the MAML_RT-based NSCLC targeted therapy efficacy prediction model, including a confusion matrix (Figure 7a) and an ROC curve (Figure 7b). In Figure 7a, for samples with a True Label of PD, the model correctly predicted 31 cases as PD, achieving a specificity of 97%, with only 1 case incorrectly predicted as PR. This shows that the model has extremely high recognition accuracy for the “Progressive Disease” category and can stably distinguish this category even in the few-shot scenario. For samples with a True Label of PR, the model correctly predicted 25 cases as PR, with a sensitivity of 89%, and 3 cases were incorrectly predicted as PD. Under the constraint of few-shot data, the model still exhibits good recognition ability for the “Partial Response” category. In Figure 7b, the AUC of the model is 0.93, which is close to 1. This indicates that under few-shot conditions, the model can efficiently distinguish between the two types of targeted therapy efficacy (PD and PR), demonstrating excellent classification and discrimination performance. Combined with the high specificity, high sensitivity from the confusion matrix results and the AUC value close to 1, the above results fully verify that the MAML_RT model has excellent classification performance in the few-shot task of NSCLC targeted therapy efficacy prediction and can reliably distinguish between the two therapeutic outcomes of PD and PR.

**FIGURE 8:**
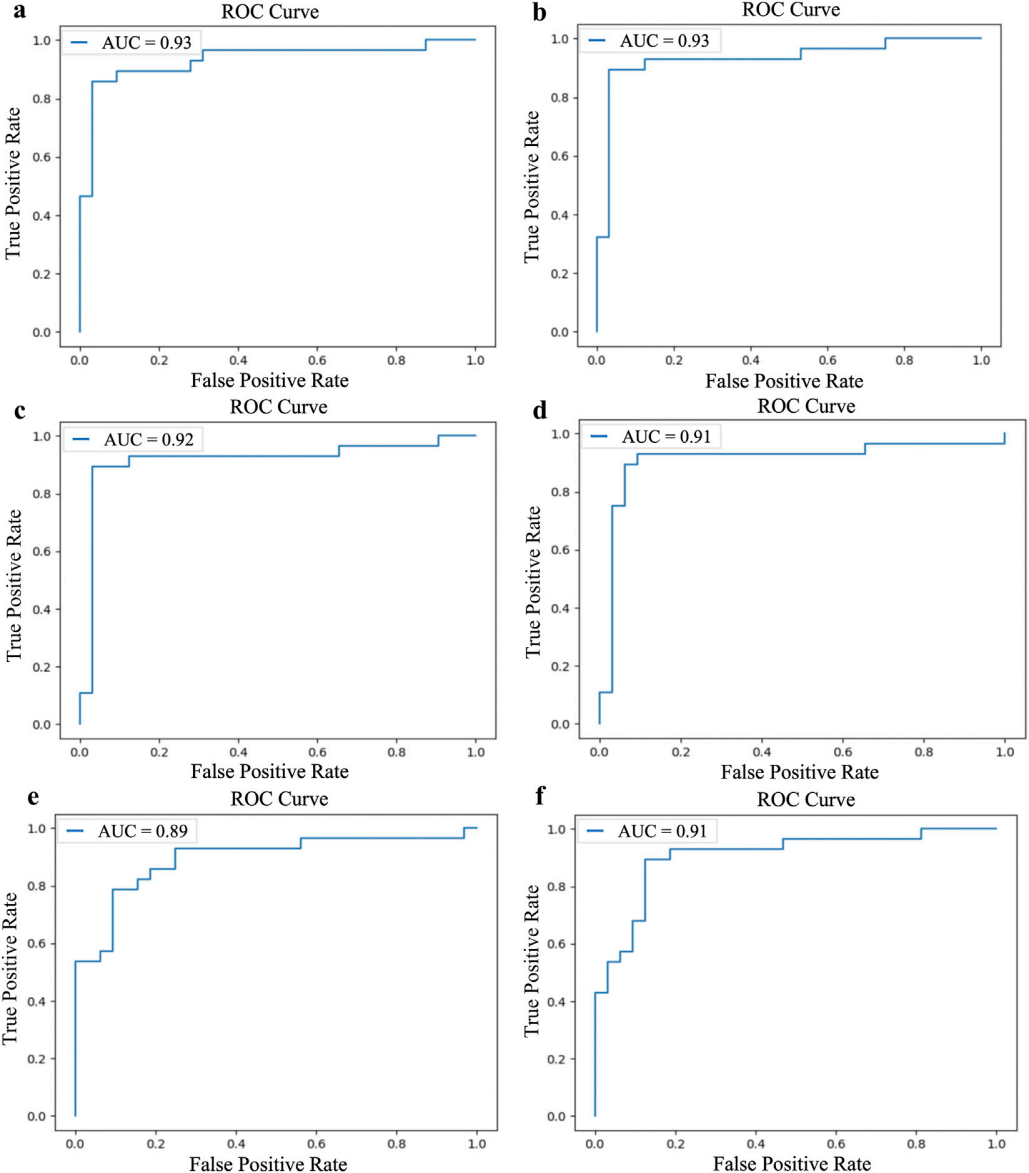
ROC curves of 6 prediction results under random sample division. **(a)**: AUC=0.93; **(b)**: AUC=0.93; **(c)**: AUC=0.92; **(d)**: AUC=0.91; **(e)**: AUC=0.89; **(f)**: AUC=0.91.

Due to the randomness of data division and potential deviations in results, six sets of results were selected as references, as shown in the Figure 8. Synthesizing all ROC curve results, the MAML_RT-based model achieved an AUC value stably above 0.89 in the few-shot NSCLC targeted therapy efficacy prediction task and even reached a high level of 0.93 in multiple trials. This result fully proves that by integrating the few-shot meta-learning capability of MAML and the feature extraction capability of Residual Transformer, the model can efficiently distinguish between the two types of targeted therapy efficacy (PD and PR) in data-scarce scenarios, providing reliable technical support for precise decision-making in NSCLC targeted therapy.

**FIGURE 9:**
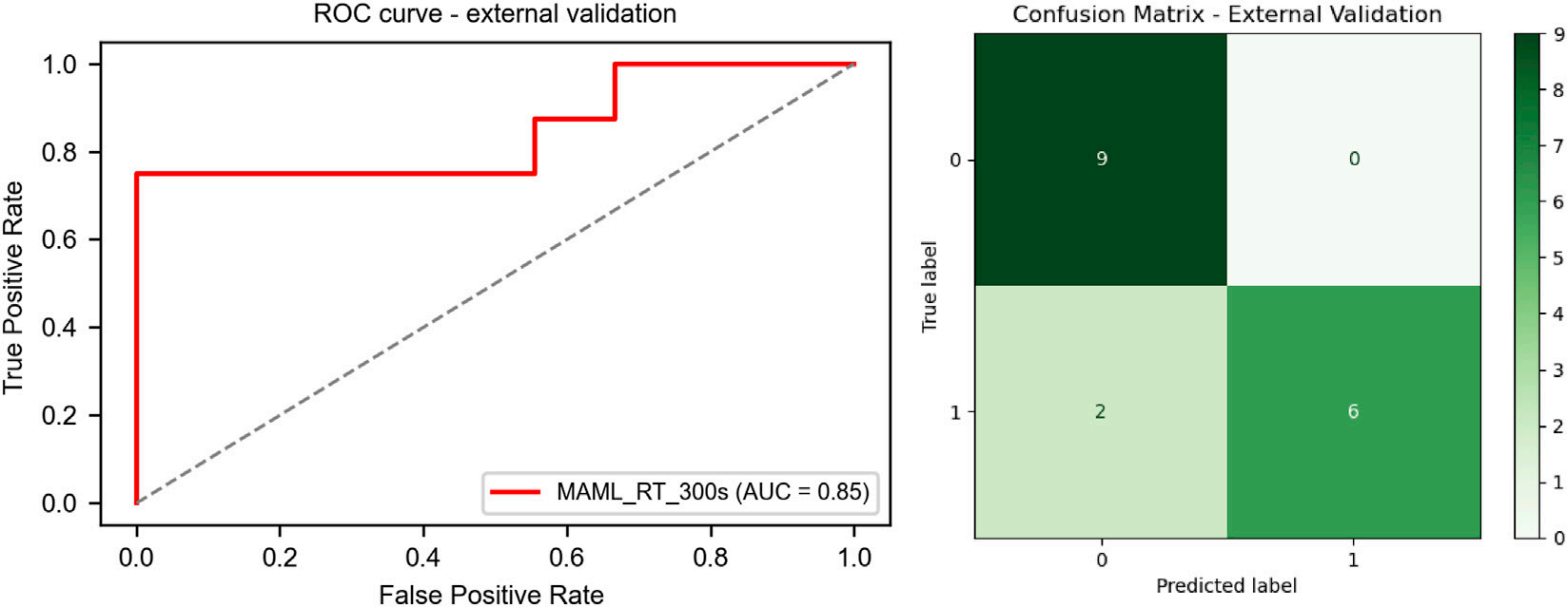
Confusion matrix and ROC curve of the external validation set.

To further evaluate the cross-center generalization capability of MAML_RT, we collected an independent external validation set from Cancer Prevention and Treatment Institute of Chengdu, Department of Oncology, Chengdu Fifth People’s Hospital, comprising 17 patients with EGFR-mutant non-small cell lung cancer, including 8 cases with PR and 9 cases with PD. This external set exhibited natural differences from the original training set in terms of CT scanning equipment, imaging parameters, geographic region, and demographic characteristics. Using the MAML_RT model trained on the original 300 cases, we performed zero-shot prediction directly on these 17 external cases (without any fine-tuning or parameter updates). The confusion matrix and ROC curve of the prediction results are shown in Figure 9. The model achieved an AUC of 0.85 and an accuracy of 0.88 on the external validation set, with a sensitivity of 1.00 and a specificity of 0.75. These results indicate that although task construction during the training phase was limited to episodic resampling within a single center, MAML_RT still maintained high discriminative performance on completely independent external center data, preliminarily validating its cross-distribution generalization potential.

## 4 Discussion

Existing research indicates that data scarcity is a key bottleneck limiting the performance of deep learning models. Particularly when dealing with rare mutations or specific populations, insufficient sample size can significantly reduce a model’s ability to generalize, making it difficult to ensure the reliability of prediction results (Bray et al., 2018). Currently, most state-of-the-art models still require 300 to 500 labeled samples to achieve stable training (Yoshioka et al., 2019), and the sample size in this study (300 cases) falls precisely at the critical threshold for performance instability. To validate the robustness of MAML_RT under decreasing data volumes, we designed Experiment A. This experiment systematically examined the performance differences between MAML_RT and the baseline model RT across varying data gradients by gradually reducing the sample size from 300 to 50.

Concurrently, to investigate the impact of feature dimension on model performance and improve computational efficiency, we conducted feature selection experiments. By analyzing the importance of different feature subsets for prediction results, we identified the most discriminative bioinformatics features, further validating the stability and generalization ability of MAML_RT in low-dimensional feature spaces.

Furthermore, the academic community has proposed various improvement strategies to address the challenge of few-shot learning. For example, Zang et al. (Wu et al., 2020) applied a visual Transformer to predict postoperative recurrence of lung cancer, achieving an AUC of 0.82 on 300 samples, but required pre-training on over 1,000 samples. Vaiyapuri et al. (2025) used variational autoencoders to generate synthetic data; Koch et al. (2015) introduced metric learning based on twin networks; and Nurgazin and Tu (2023) utilized the Swin Transformer to enhance feature extraction. All these methods have demonstrated potential for addressing data scarcity in medical applications. However, the performance of these methods on the specific task of predicting the efficacy of targeted therapy for non-small cell lung cancer still requires further validation. To this end, we designed Experiment B to compare the predictive performance of MAML_RT with the aforementioned mainstream few-shot learning models under extremely few-shot conditions (50 samples), as well as with three strong tabular baseline models.

Furthermore, to comprehensively evaluate the advantages of MAML_RT under different training paradigms, we designed comparative experiments to systematically compare the performance of Joint Learning and Transfer Learning on this task, thereby revealing the unique value of the meta-learning paradigm in low-data scenarios.

Through the longitudinal evolution in Experiment A, dimensional analysis in the feature selection experiments, horizontal competition in Experiment B, and the comparison of Joint Learning and Transfer Learning paradigms, this paper aims to comprehensively evaluate the combined advantages of MAML_RT in terms of “low-data efficiency” and “feature representation capability.”

### 4.1 ROC comparison of prediction performance between MAML_RT and RT under different sample sizes

**FIGURE 10:**
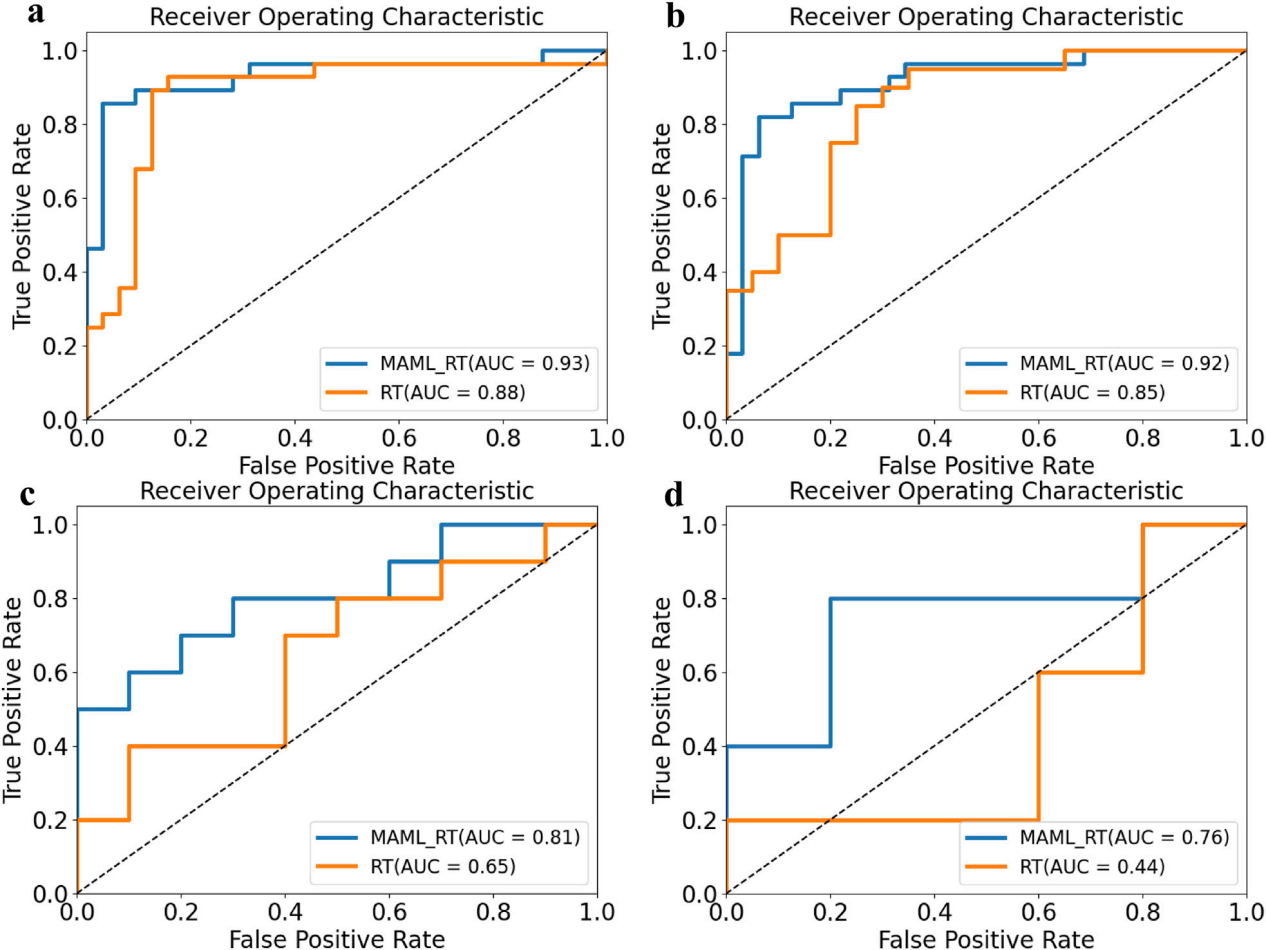
ROC Comparison of Prediction Performance Between MAML_RT and RT Under Different Sample Sizes: **(a)** 300 samples, **(b)** 200 samples, **(c)** 100 samples, **(d)** 50 samples.

By comparing the ROC curves and AUC values of MAML_RT against the residual transformer baseline model across different sample sizes, this study thoroughly investigates the advantages, underlying mechanisms, and clinical significance of the meta-learning architecture in predicting the efficacy of targeted therapies for NSCLC with limited samples. As demonstrated by four comparative experiments (seeing Figure 10), MAML_RT consistently outperformed the RT model regardless of sample size: achieving an AUC of 0.93 (RT: 0.88) with 300 samples, 0.92 (RT: 0.85) with 200 samples, even at 100 samples it maintained 0.81 (RT only 0.65), and when the sample size was drastically reduced to 50, MAML_RT still achieved an AUC of 0.76, while RT dropped to 0.44. This significant performance gap stems from MAML’s meta-learning mechanism—by undergoing “meta-training” across multiple few-shot tasks, the model acquires cross-task general knowledge. This includes universal association patterns between NSCLC targeted therapy efficacy and molecular/imaging features, enabling rapid adaptation and generalization in the target few-shot prediction task. In contrast, the baseline model RT, which possesses only feature extraction capabilities, lacks targeted optimization for few-shot scenarios, leading to a steep decline in performance as sample size decreases. These experimental results validate the necessity of integrating meta-learning with the Residual Transformer deep architecture and provide an algorithmic explanation for MAML_RT’s performance advantage in data-scarce scenarios.

As the sample size gradually decreased from 300 to 50, both MAML_RT and RT exhibited declining AUC values. However, MAML_RT demonstrated a more gradual performance degradation, revealing superior robustness. Quantitative analysis reveals that when the sample size shrinks by approximately 83% (from 300 to 50), MAML_RT’s AUC decreases from 0.93 to 0.76, representing an 18.3% decline. In contrast, RT’s AUC plummets from 0.88 to 0.44, a staggering 50% drop. This demonstrates that its meta-learning mechanism effectively mitigates the “data scarcity-induced overfitting” problem common in medical image analysis, enabling the model to maintain acceptable predictive performance even with extremely small sample sizes. This characteristic holds significant practical value for NSCLC targeted therapy clinical practice: in real-world settings, acquiring large-scale, well-annotated case data is highly challenging due to constraints in medical resource allocation, the invasive risks of needle biopsy, patient compliance, and disease progression velocity. Consequently, the need for analysis of extremely small or even minuscule sample sizes is extremely common. The robustness demonstrated by MAML_RT enables it to provide reliable technical support for clinical decision-making (such as early efficacy assessment and dynamic treatment plan adjustments) within these constrained scenarios.

**FIGURE 11:**
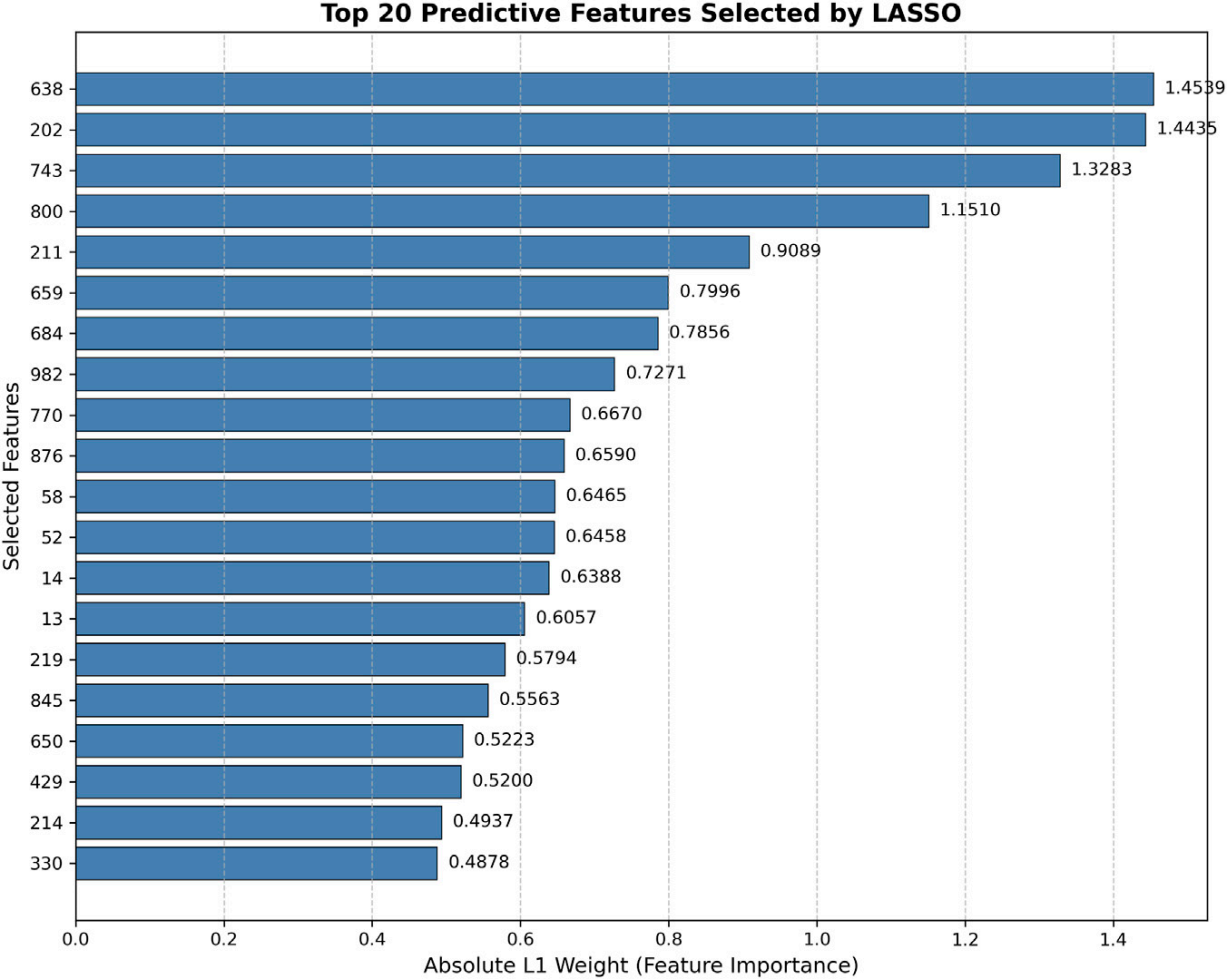
Top 20 radiomics features with the highest predictive efficacy selected by LASSO regression and their corresponding regression coefficients.

**FIGURE 12:**
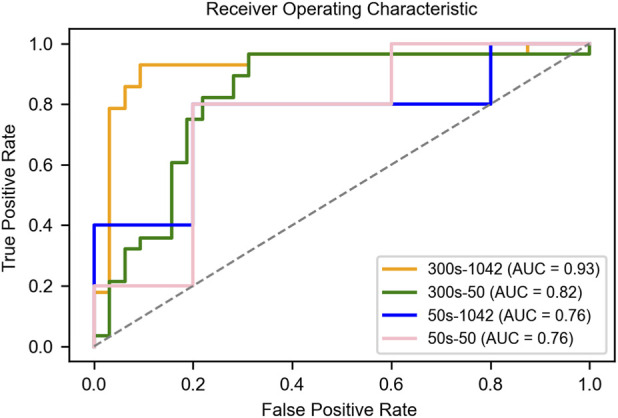
Comparison of ROC curves of MAML_RT under different sample sizes (300/50) and feature quantities (full 1042/top 50 selected by LASSO).

To quantitatively assess the actual impact of high-dimensional features on model stability and overfitting, we used LASSO regression to rank the importance of all 1,042 features. The top 20 features with the highest predictive power and their coefficients are shown Figure 11. Furthermore, we compared the predictive performance of MAML_RT under two conditions—300 samples and 50 samples—when using all 1,042 features versus only the top 50 features selected via LASSO. The corresponding ROC curves and AUC values are summarized in Figure 12. Under the 300-sample condition, the AUC was 0.93 when using all features, while it dropped to 0.82 when using only the 50 filtered features. This indicates that the full-feature model did not overfit within this framework; rather, it achieved superior performance by retaining more weakly correlated features that exhibit synergistic predictive effects. Under the extreme condition of 50 samples, the AUC for both the full-feature model and the filtered-feature model is 0.76. This indicates that when the sample size is extremely scarce, model performance is constrained by the number of samples rather than the feature dimension, and high-dimensional features do not cause a collapse in performance. In summary, the MAML_RT framework, combined with an explicit regularization strategy, effectively addresses the overfitting risks associated with high-dimensional, small-sample datasets. The full-feature model, which does not perform active feature selection, performs best under the 300-sample condition.

### 4.2 Comparison with few-shot and tabular baseline models

**FIGURE 13:**
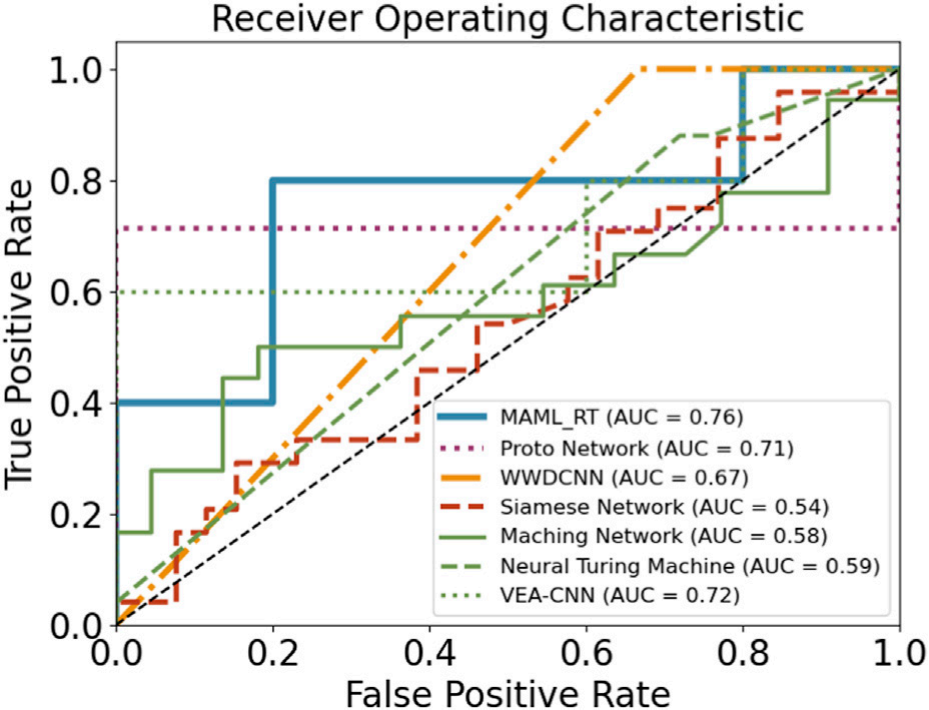
Comparison of prediction performance among different models with 50 samples.

As shown in Figure 13, MAML_RT demonstrates significant performance advantages even under the extreme condition of only 50 samples. Specifically, its AUC value of 0.76 markedly surpasses other mainstream comparison models. Within the meta-learning paradigm, MAML_RT outperforms Proto Network and VEA-CNN, which yielded AUC values of 0.71 and 0.72, respectively. The performance gap is even more pronounced when compared to traditional deep learning and metric learning models, such as WWDCNN with an AUC of 0.67, Matching Network at 0.58, and Siamese Network at 0.54. Although Proto Network and VEA-CNN also follow few-shot meta-learning principles, MAML_RT achieves superior predictive accuracy. This advantage originates from the meta-learning strategy based on gradient descent, which facilitates finer-grained optimization for model parameter updates. In contrast to the prototype matching used in Proto Network or the variational autoencoder architecture in VEA-CNN, the MAML_RT framework demonstrates higher adaptability for complex medical tasks. Conversely, models like WWDCNN and Siamese networks rely heavily on conventional feature extraction or metric learning. These approaches often fail to capture task-general knowledge in data-scarce environments, leading to issues with underfitting or overfitting. Consequently, their AUC values remain significantly lower than that of MAML_RT. These findings validate the critical role of meta-learning in few-shot medical prediction and underscore the technical advancement of the MAML_RT architecture.

**FIGURE 14:**
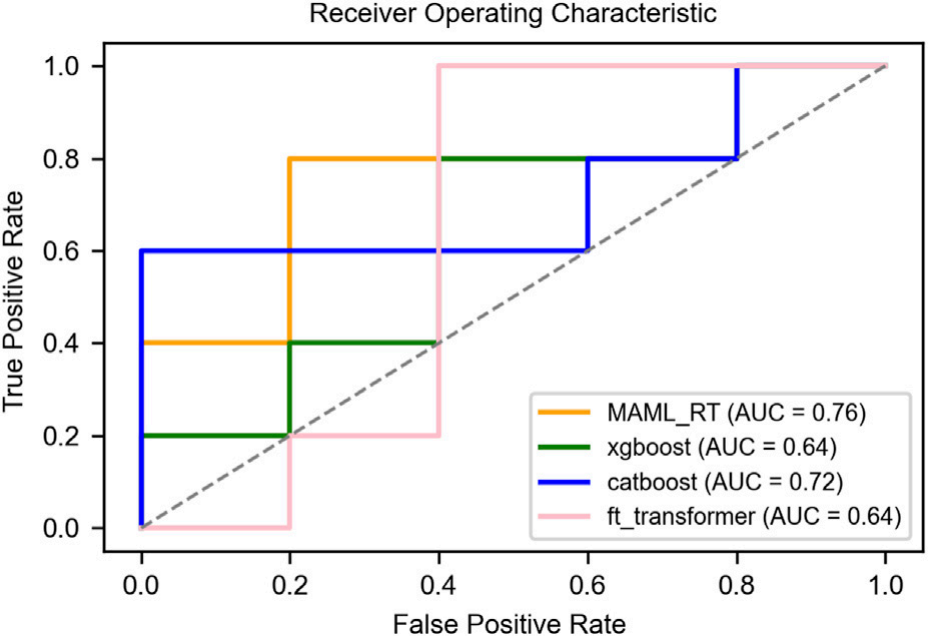
Comparison of ROC curves of MAML_RT versus XGBoost, CatBoost, and FT-Transformer under the condition of 50 samples.

Furthermore, considering that radiomics features are essentially tabular data, we further compared MAML_RT with three strong tabular baseline models under the extreme condition of 50 samples, including the gradient boosting tree-based XGBoost and CatBoost, as well as FT-Transformer, a model specifically designed for tabular data. As shown in Figure 14, MAML_RT achieved an AUC of 0.76, whereas XGBoost, CatBoost, and FT-Transformer achieved AUCs of 0.64, 0.72, and 0.64, respectively. CatBoost performed relatively best among the tabular models (0.72), yet it was still significantly inferior to MAML_RT. These results indicate that when the sample size is extremely scarce, conventional tabular models, due to their lack of cross-task transfer learning capabilities, tend to suffer from overfitting or fail to adequately exploit feature interactions. In contrast, MAML_RT, through its learnable generalizable initial parameters obtained via meta-learning, combined with the residual Transformer’s ability to model global dependencies among features, can more efficiently extract discriminative signals from limited samples, thereby demonstrating superior few-shot prediction performance.

The results above demonstrate that MAML’s model-agnostic meta-learning framework enables models to learn how to rapidly adapt to new tasks across multiple tasks. In the target task of predicting the efficacy of targeted therapies for NSCLC, this framework allows the model to generalize quickly with only a small number of samples, effectively overcoming the overfitting problem in small-sample scenarios. Simultaneously, the residual Transformer demonstrates advantages in multimodal feature interaction and modeling long-range dependencies, efficiently integrating multi-source data such as gene mutations, imaging features, and clinical indicators to extract more discriminative features. The fusion of these two components enables MAML_RT to achieve dual breakthroughs in both few-shot learning efficiency and medical feature expression capabilities, providing a reliable solution for efficient prediction using limited clinical data.

### 4.3 Comparison with joint training and transfer learning paradigms

To further validate the effectiveness of the proposed MAML_RT framework in subgroup knowledge transfer, this study selected Joint Training and Transfer Learning as representative baseline models for comparative analysis.

**FIGURE 15:**
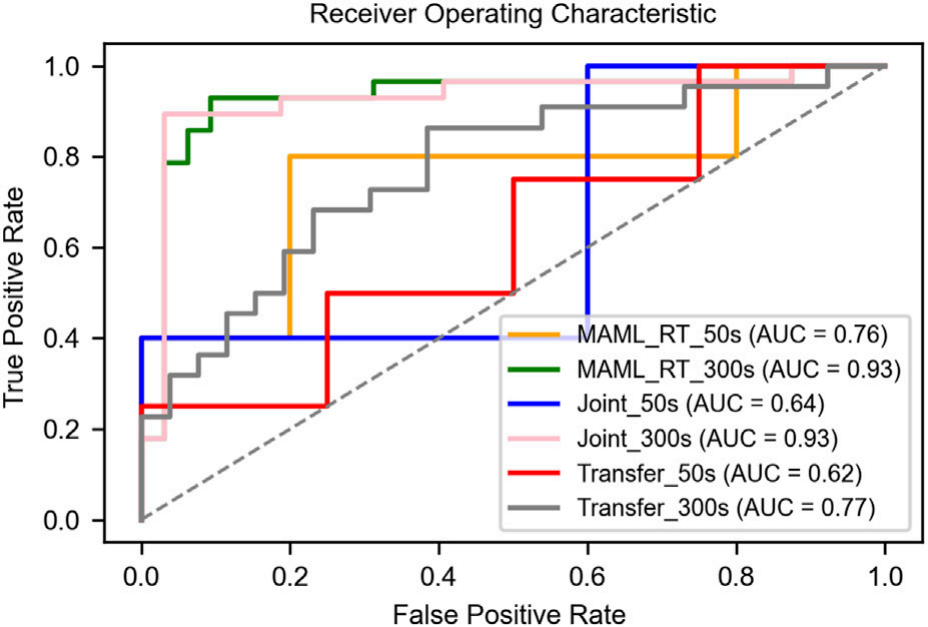
Comparison of AUC of MAML_RT versus joint training and transfer learning under different sample sizes (300/50) using leave-one-subgroup-out validation.

First, we conduct a comparison in a single-center internal validation scenario. As shown in Figure 15, when using all 300 training samples, both MAML_RT and Joint Training achieve an AUC of approximately 0.93, while Transfer Learning achieves an AUC of 0.77, indicating that when samples are relatively abundant, both meta-learning and traditional supervised learning can achieve good predictive performance. However, in a very low-sample-size scenario using only 50 training samples, MAML_RT still maintains an AUC of 0.76, while joint training and transfer learning drop to 0.64 and 0.62, respectively. The results indicate that traditional supervised learning methods are more prone to overfitting under high-dimensional, low-sample conditions, whereas meta-learning learns parameter initializations with greater generalization ability through a two-layer optimization mechanism, thereby demonstrating stronger adaptability to low-sample scenarios.

**FIGURE 16:**
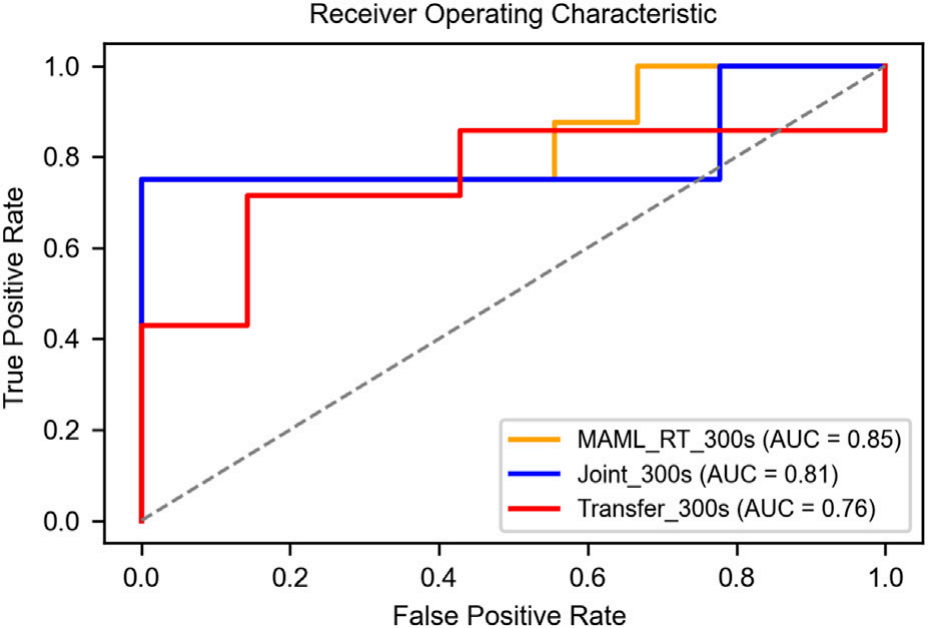
Comparison of generalization performance of different models on the external validation set.

Furthermore, to evaluate the model’s cross-domain generalization capability, this study adopted a Leave-One-Center-Out validation strategy, using 300 patients from Dongyang People’s Hospital as the training set and 17 patients from Chengdu Fifth People’s Hospital as a completely independent external test set. Experimental results show that MAML_RT achieves an AUC of 0.85 on the external center, outperforming joint training (0.81) and transfer learning (0.76) (Figure 16). Since the two centers exhibit significant differences in terms of scanning equipment, imaging parameters, geographic origin, and patient characteristics, this experiment constitutes a rigorous cross-domain validation scenario. The results indicate that MAML_RT can learn domain-invariant knowledge representations, thereby achieving superior generalization performance on unseen external subgroups.

### 4.4 Limitations and future work

The MAML_RT framework delivers significant performance advantages under few-shot constraints. However, when the sample size is drastically reduced (e.g., n = 50), the model’s predictive performance (AUC = 0.76) surpasses baseline methods yet does not meet the stringent clinical demand for “ultra-high-precision prediction in ultra-small-sample scenarios.” To overcome this bottleneck, future improvements will focus on the following directions:

1. Integration of data augmentation and meta-learning: Introduce data augmentation technologies suitable for the medical field (e.g., generative adversarial network-based augmentation for imaging features, virtual sample generation for omics features) to provide more abundant “pseudo-samples” for meta-learning, further improving performance under extremely small sample sizes.
2. Classification expansion: This study only focuses on the binary classification task of “PR vs. PD”. In the future, it can be extended to a multi-classification scenario including “Complete Response (CR), Partial Response (PR), Stable Disease (SD), and Progressive Disease (PD)”. Meanwhile, classification can be further refined based on the types of targeted drugs, expanding to multi-classification scenarios for the efficacy of different targeted drugs to further improve the clinical applicability of the model.
3. Acknowledge the limitations of this study regarding the design of meta-learning tasks: we only performed random segment-based resampling within a single-center cohort and failed to introduce subgroups that truly span different distributions (such as different mutation subtypes) during the training phase. This approach to task design limits the model’s robustness to distribution shifts. Although independent external validation results (AUC = 0.85) indicate that the model still possesses some cross-center generalization capability, future research needs to develop meta-tasks that truly encompass different subgroups to fully realize MAML’s potential for domain generalization.
4. Enhancing model interpretability: This study primarily focuses on model predictive performance; however, the exploration of the model’s internal decision-making mechanisms and their biological significance remains relatively limited. In the future, we plan to incorporate interpretability methods such as SHAP and attention weight analysis to identify and quantify the key features of interest in the model. By integrating multimodal information from fields such as pathology and genomics, we aim to explore potential associations between imaging features and the efficacy of EGFR-TKIs as well as mechanisms of drug resistance, thereby enhancing the model’s interpretability and clinical credibility.

## 5 Conclusion

To address the common issue of insufficient sample size in current research, this paper proposes the MAML_RT model. At its core, this model adopts the MAML paradigm. Through a multi-task meta-training stage, it acquires initial weights with high task generalization potential. This enables rapid convergence via efficient gradient iteration even with minimal samples, significantly reducing deep learning prediction models’ excessive reliance on massive labeled datasets. At the feature extraction level, the RT models global correlations among high-dimensional radiomics features through self-attention mechanisms. This effectively captures long-range dependencies and latent interactions between different imaging textures, shapes, and intensity distribution metrics, thereby addressing the limitations of traditional convolutional or fully connected architectures in modeling complex relationships between features.

In a test cohort of 300 patients with non-small cell lung cancer, the MAML_RT model demonstrated exceptional performance in predicting responses to targeted therapy: on the internal test set, it achieved an AUC of 0.93, a precision of 0.91, a sensitivity of 0.89, and a specificity of 0.97; on the external validation set, the AUC was 0.85, the accuracy was 0.88, a sensitivity of 1.00. The model demonstrated high reliability in distinguishing between the two clinical outcomes of PD and PR. Ablation studies showed that while the performance of all models fluctuated with decreasing training sample size, MAML_RT consistently outperformed the single residual Transformer architecture and exhibited significantly lower sensitivity to data reduction. In experiments with a very small sample size (n = 50), MAML_RT achieved an AUC of 0.76, outperforming ProtoNet (0.71) and VEA-CNN (0.72), making it one of the few models capable of maintaining clinical relevance under extremely limited data constraints. These results fully demonstrate that MAML_RT achieves a good balance between “learning efficiency with few samples” and “the ability to capture complex medical features,” providing robust technical support for precision medicine in data-scarce scenarios.

## Data Availability

The raw data supporting the conclusions of this article will be made available by the authors, without undue reservation.
